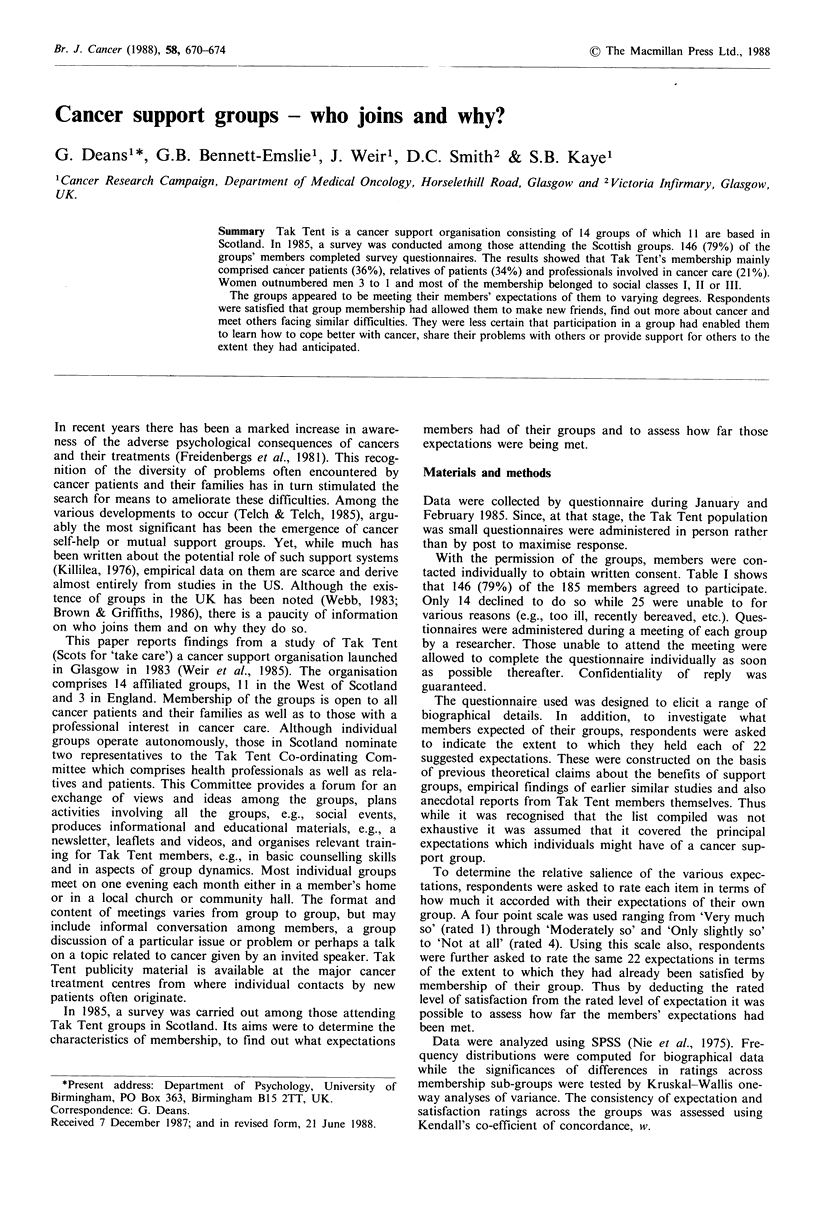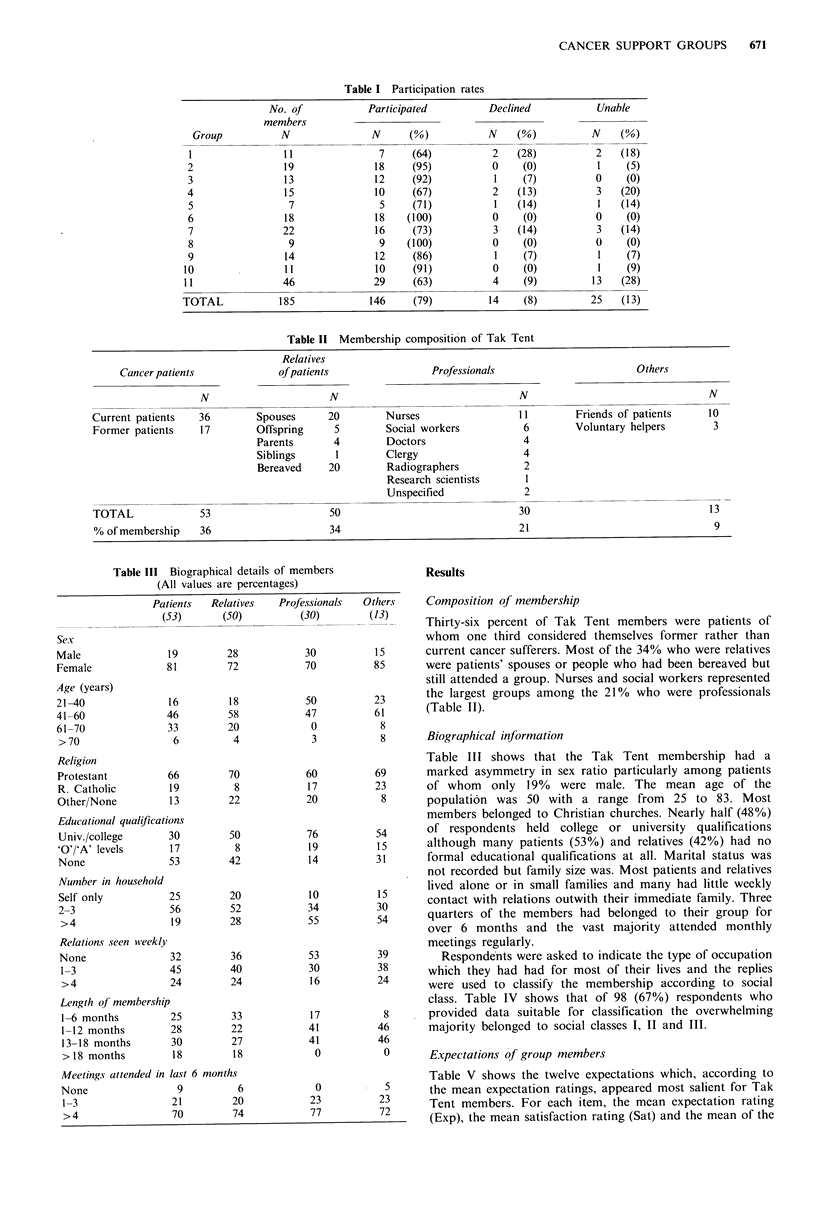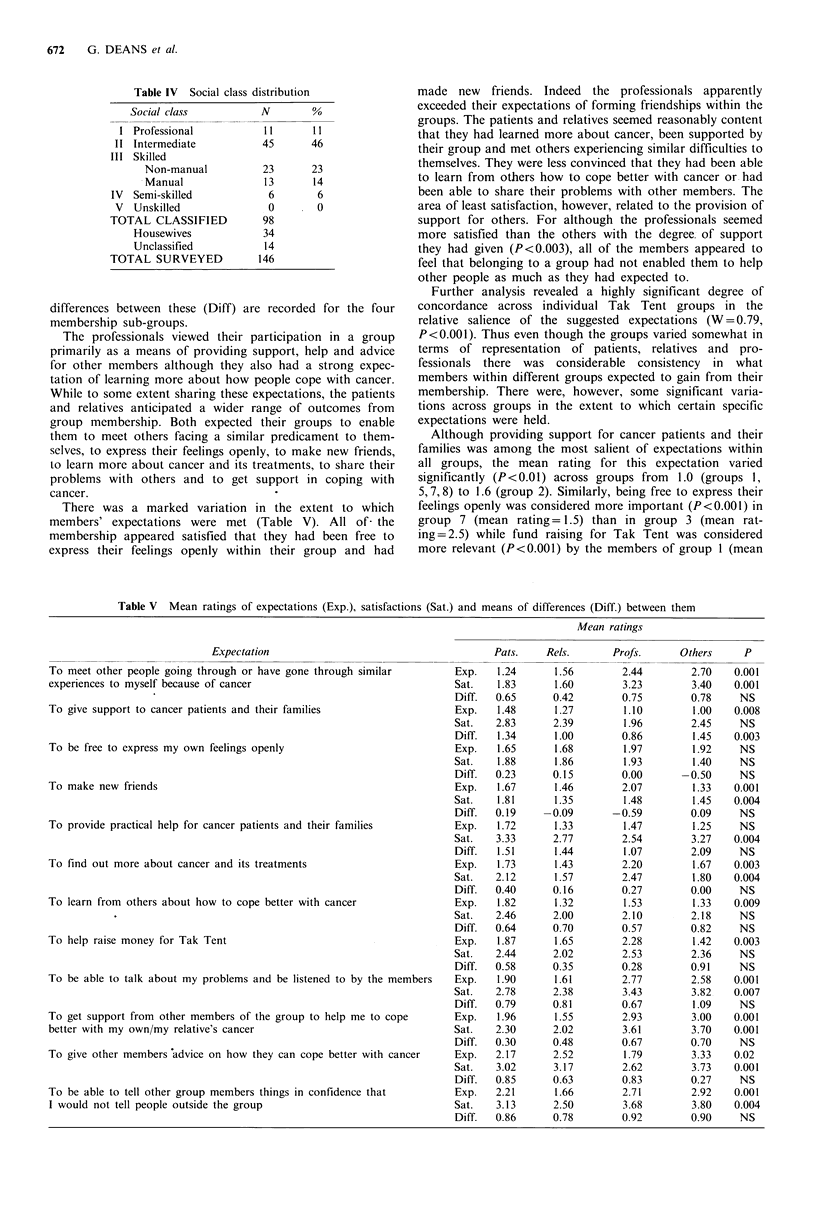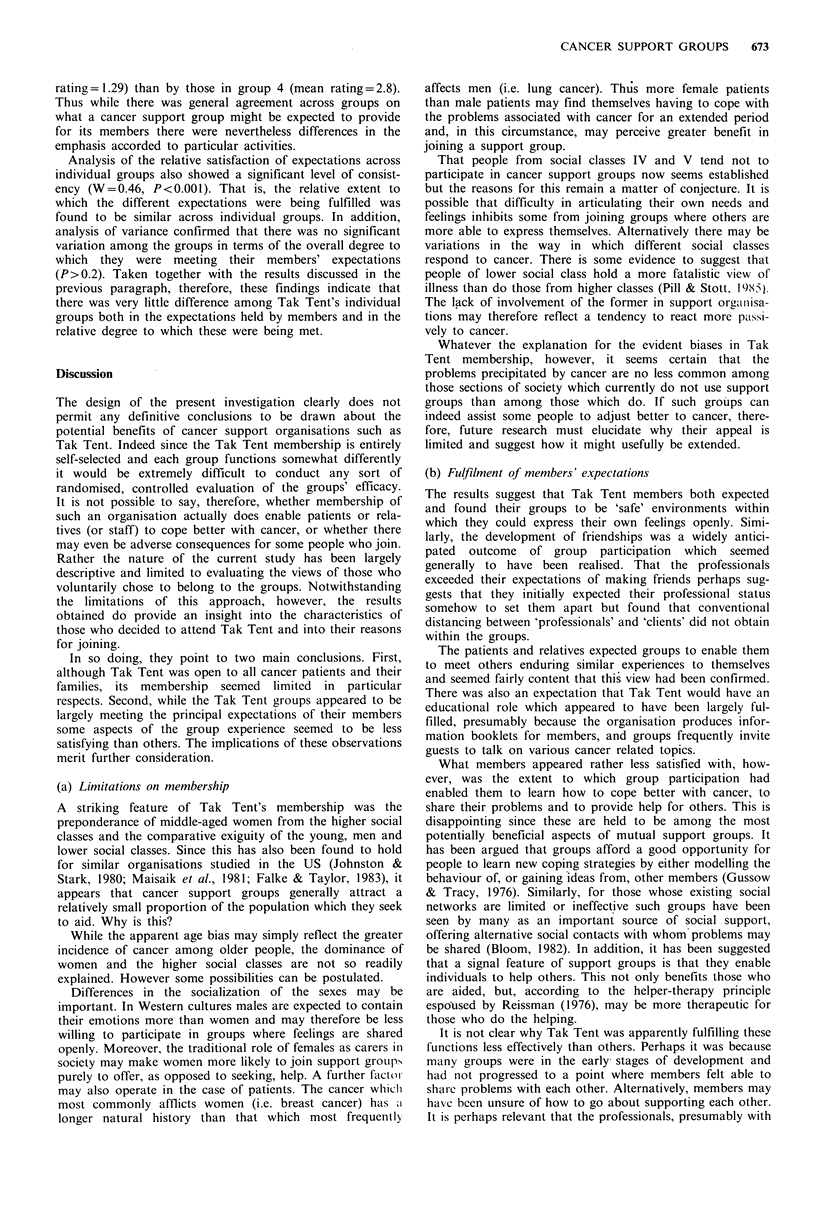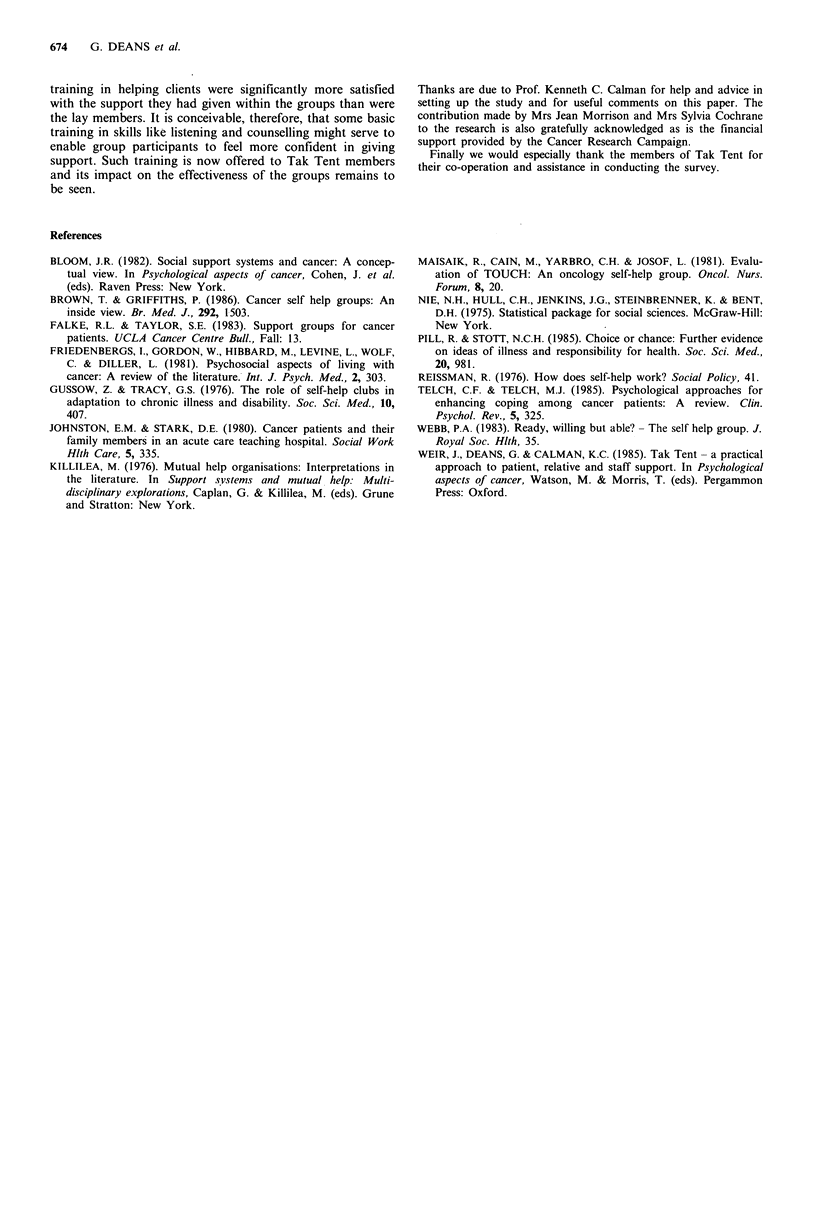# Cancer support groups--who joins and why?

**DOI:** 10.1038/bjc.1988.284

**Published:** 1988-11

**Authors:** G. Deans, G. B. Bennett-Emslie, J. Weir, D. C. Smith, S. B. Kaye

**Affiliations:** Cancer Research Campaign, Department of Medical Oncology, Glasgow, UK.

## Abstract

Tak Tent is a cancer support organisation consisting of 14 groups of which 11 are based in Scotland. In 1985, a survey was conducted among those attending the Scottish groups. 146 (79%) of the groups' members completed survey questionnaires. The results showed that Tak Tent's membership mainly comprised cancer patients (36%), relatives of patients (34%) and professionals involved in cancer care (21%). Women outnumbered men 3 to 1 and most of the membership belonged to social classes I, II or III. The groups appeared to be meeting their members' expectations of them to varying degrees. Respondents were satisfied that group membership had allowed them to make new friends, find out more about cancer and meet others facing similar difficulties. They were less certain that participation in a group had enabled them to learn how to cope better with cancer, share their problems with others or provide support for others to the extent they had anticipated.


*Br. J. Cancer* (1988), **58**, 670–674 © The Macmillan Press Ltd., 1988

### Cancer support groups – who joins and why?

G. Deans[1]*, G.B. Bennett-Emslie[1], J. Weir[1], D.C. Smith[2] & S.B. Kaye[1]

[1]*Cancer Research Campaign, Department of Medical Oncology, Horselethill Road, Glasgow and* [2]*Victoria Infirmary, Glasgow, UK.*

**Summary** Tak Tent is a cancer support organisation consisting of 14 groups of which 11 are based in Scotland. In 1985, a survey was conducted among those attending the Scottish groups. 146 (79%) of the groups' members completed survey questionnaires. The results showed that Tak Tent's membership mainly comprised cancer patients (36%), relatives of patients (34%) and professionals involved in cancer care (21%). Women outnumbered men 3 to 1 and most of the membership belonged to social classes I, II or III.

The groups appeared to be meeting their members' expectations of them to varying degrees. Respondents were satisfied that group membership had allowed them to make new friends, find out more about cancer and meet others facing similar difficulties. They were less certain that participation in a group had enabled them to learn how to cope better with cancer, share their problems with others or provide support for others to the extent they had anticipated.

In recent years there has been a marked increase in awareness of the adverse psychological consequences of cancers and their treatments (Freidenbergs *et al*., 1981). This recognition of the diversity of problems often encountered by cancer patients and their families has in turn stimulated the search for means to ameliorate these difficulties. Among the various developments to occur (Telch & Telch, 1985), arguably the most significant has been the emergence of cancer self-help or mutual support groups. Yet, while much has been written about the potential role of such support systems (Killilea, 1976), empirical data on them are scarce and derive almost entirely from studies in the US. Although the existence of groups in the UK has been noted (Webb, 1983; Brown & Griffiths, 1986), there is a paucity of information on who joins them and on why they do so.

This paper reports findings from a study of Tak Tent (Scots for 'take care') a cancer support organisation launched in Glasgow in 1983 (Weir *et al*., 1985). The organisation comprises 14 affiliated groups, 11 in the West of Scotland and 3 in England. Membership of the groups is open to all cancer patients and their families as well as to those with a professional interest in cancer care. Although individual groups operate autonomously, those in Scotland nominate two representatives to the Tak Tent Co-ordinating Committee which comprises health professionals as well as relatives and patients. This Committee provides a forum for an exchange of views and ideas among the groups, plans activities involving all the groups, e.g., social events, produces informational and educational materials, e.g., a newsletter, leaflets and videos, and organises relevant training for Tak Tent members, e.g., in basic counselling skills and in aspects of group dynamics. Most individual groups meet on one evening each month either in a member's home or in a local church or community hall. The format and content of meetings varies from group to group, but may include informal conversation among members, a group discussion of a particular issue or problem or perhaps a talk on a topic related to cancer given by an invited speaker. Tak Tent publicity material is available at the major cancer treatment centres from where individual contacts by new patients often originate.

In 1985, a survey was carried out among those attending Tak Tent groups in Scotland. Its aims were to determine the characteristics of membership, to find out what expectations members had of their groups and to assess how far those expectations were being met.

*Present address: Department of Psychology, University of Birmingham, PO Box 363, Birmingham B15 2TT, UK.
Correspondence: G. Deans.
Received 7 December 1987; and in revised form, 21 June 1988.

### Materials and methods

Data were collected by questionnaire during January and February 1985. Since, at that stage, the Tak Tent population was small questionnaires were administered in person rather than by post to maximise response.

With the permission of the groups, members were contacted individually to obtain written consent. Table I shows that 146 (79%) of the 185 members agreed to participate. Only 14 declined to do so while 25 were unable to for various reasons (e.g., too ill, recently bereaved, etc.). Questionnaires were administered during a meeting of each group by a researcher. Those unable to attend the meeting were allowed to complete the questionnaire individually as soon as possible thereafter. Confidentiality of reply was guaranteed.

The questionnaire used was designed to elicit a range of biographical details. In addition, to investigate what members expected of their groups, respondents were asked to indicate the extent to which they held each of 22 suggested expectations. These were constructed on the basis of previous theoretical claims about the benefits of support groups, empirical findings of earlier similar studies and also anecdotal reports from Tak Tent members themselves. Thus while it was recognised that the list compiled was not exhaustive it was assumed that it covered the principal expectations which individuals might have of a cancer support group.

To determine the relative salience of the various expectations, respondents were asked to rate each item in terms of how much it accorded with their expectations of their own group. A four point scale was used ranging from 'Very much so' (rated 1) through 'Moderately so' and 'Only slightly so' to 'Not at all' (rated 4). Using this scale also, respondents were further asked to rate the same 22 expectations in terms of the extent to which they had already been satisfied by membership of their group. Thus by deducting the rated level of satisfaction from the rated level of expectation it was possible to assess how far the members' expectations had been met.

Data were analyzed using SPSS (Nie *et al*., 1975). Frequency distributions were computed for biographical data while the significances of differences in ratings across membership sub-groups were tested by Kruskal–Wallis one-way analyses of variance. The consistency of expectation and satisfaction ratings across the groups was assessed using Kendall's co-efficient of concordance, *w*.

**Table I** Participation rates

| Group | No. of members N | Participated N | (%) | Declined N | (%) | Unable N | (%) |
|---|---|---|---|---|---|---|---|
| 1 | 11 | 7 | (64) | 2 | (28) | 2 | (18) |
| 2 | 19 | 18 | (95) | 0 | (0) | 1 | (5) |
| 3 | 13 | 12 | (92) | 1 | (7) | 0 | (0) |
| 4 | 15 | 10 | (67) | 2 | (13) | 3 | (20) |
| 5 | 7 | 5 | (71) | 1 | (14) | 1 | (14) |
| 6 | 18 | 18 | (100) | 0 | (0) | 0 | (0) |
| 7 | 22 | 16 | (73) | 3 | (14) | 3 | (14) |
| 8 | 9 | 9 | (100) | 0 | (0) | 0 | (0) |
| 9 | 14 | 12 | (86) | 1 | (7) | 1 | (7) |
| 10 | 11 | 10 | (91) | 0 | (0) | 1 | (9) |
| 11 | 46 | 29 | (63) | 4 | (9) | 13 | (28) |
| TOTAL | 185 | 146 | (79) | 14 | (8) | 25 | (13) |

**Table II** Membership composition of Tak Tent

| Cancer patients | N | Relatives of patients | N | Professionals | N | Others | N |
|---|---|---|---|---|---|---|---|
| Current patients | 36 | Spouses | 20 | Nurses | 11 | Friends of patients | 10 |
| Former patients | 17 | Offspring | 5 | Social workers | 6 | Voluntary helpers | 3 |
| | | Parents | 4 | Doctors | 4 | | |
| | | Siblings | 1 | Clergy | 4 | | |
| | | Bereaved | 20 | Radiographers | 2 | | |
| | | | | Research scientists | 1 | | |
| | | | | Unspecified | 2 | | |
| TOTAL | 53 | | 50 | | 30 | | 13 |
| % of membership | 36 | | 34 | | 21 | | 9 |

**Table III** Biographical details of members (All values are percentages)

| | Patients (53) | Relatives (50) | Professionals (30) | Others (13) |
|---|---|---|---|---|
| *Sex* | | | | |
| Male | 19 | 28 | 30 | 15 |
| Female | 81 | 72 | 70 | 85 |
| *Age* (years) | | | | |
| 21–40 | 16 | 18 | 50 | 23 |
| 41–60 | 46 | 58 | 47 | 61 |
| 61–70 | 33 | 20 | 0 | 8 |
| >70 | 6 | 4 | 3 | 8 |
| *Religion* | | | | |
| Protestant | 66 | 70 | 60 | 69 |
| R. Catholic | 19 | 8 | 17 | 23 |
| Other/None | 13 | 22 | 20 | 8 |
| *Educational qualifications* | | | | |
| Univ./college | 30 | 50 | 76 | 54 |
| 'O'/'A' levels | 17 | 8 | 19 | 15 |
| None | 53 | 42 | 14 | 31 |
| *Number in household* | | | | |
| Self only | 25 | 20 | 10 | 15 |
| 2–3 | 56 | 52 | 34 | 30 |
| >4 | 19 | 28 | 55 | 54 |
| *Relations seen weekly* | | | | |
| None | 32 | 36 | 53 | 39 |
| 1–3 | 45 | 40 | 30 | 38 |
| >4 | 24 | 24 | 16 | 24 |
| *Length of membership* | | | | |
| 1–6 months | 25 | 33 | 17 | 8 |
| 1–12 months | 28 | 22 | 41 | 46 |
| 13–18 months | 30 | 27 | 41 | 46 |
| >18 months | 18 | 18 | 0 | 0 |
| *Meetings attended in last 6 months* | | | | |
| None | 9 | 6 | 0 | 5 |
| 1–3 | 21 | 20 | 23 | 23 |
| >4 | 70 | 74 | 77 | 72 |

### Results

### *Composition of membership*

Thirty-six percent of Tak Tent members were patients of whom one third considered themselves former rather than current cancer sufferers. Most of the 34% who were relatives were patients' spouses or people who had been bereaved but still attended a group. Nurses and social workers represented the largest groups among the 21% who were professionals (Table II).

### *Biographical information*

Table III shows that the Tak Tent membership had a marked asymmetry in sex ratio particularly among patients of whom only 19% were male. The mean age of the population was 50 with a range from 25 to 83. Most members belonged to Christian churches. Nearly half (48%) of respondents held college or university qualifications although many patients (53%) and relatives (42%) had no formal educational qualifications at all. Marital status was not recorded but family size was. Most patients and relatives lived alone or in small families and many had little weekly contact with relations outwith their immediate family. Three quarters of the members had belonged to their group for over 6 months and the vast majority attended monthly meetings regularly.

Respondents were asked to indicate the type of occupation which they had had for most of their lives and the replies were used to classify the membership according to social class. Table IV shows that of 98 (67%) respondents who provided data suitable for classification the overwhelming majority belonged to social classes I, II and III.

### *Expectations of group members*

Table V shows the twelve expectations which, according to the mean expectation ratings, appeared most salient for Tak Tent members. For each item, the mean expectation rating (Exp), the mean satisfaction rating (Sat) and the mean of the

**Table IV** Social class distribution

| Social class | N | % |
|---|---|---|
| I Professional | 11 | 11 |
| II Intermediate | 45 | 46 |
| III Skilled | | |
| Non-manual | 23 | 23 |
| Manual | 13 | 14 |
| IV Semi-skilled | 6 | 6 |
| V Unskilled | 0 | 0 |
| TOTAL CLASSIFIED | 98 | |
| Housewives | 34 | |
| Unclassified | 14 | |
| TOTAL SURVEYED | 146 | |

differences between these (Diff) are recorded for the four membership sub-groups.

The professionals viewed their participation in a group primarily as a means of providing support, help and advice for other members although they also had a strong expectation of learning more about how people cope with cancer. While to some extent sharing these expectations, the patients and relatives anticipated a wider range of outcomes from group membership. Both expected their groups to enable them to meet others facing a similar predicament to themselves, to express their feelings openly, to make new friends, to learn more about cancer and its treatments, to share their problems with others and to get support in coping with cancer.

There was a marked variation in the extent to which members' expectations were met (Table V). All of the membership appeared satisfied that they had been free to express their feelings openly within their group and had made new friends. Indeed the professionals apparently exceeded their expectations of forming friendships within the groups. The patients and relatives seemed reasonably content that they had learned more about cancer, been supported by their group and met others experiencing similar difficulties to themselves. They were less convinced that they had been able to learn from others how to cope better with cancer or had been able to share their problems with other members. The area of least satisfaction, however, related to the provision of support for others. For although the professionals seemed more satisfied than the others with the degree of support they had given ($P<0.003$), all of the members appeared to feel that belonging to a group had not enabled them to help other people as much as they had expected to.

Further analysis revealed a highly significant degree of concordance across individual Tak Tent groups in the relative salience of the suggested expectations ($W=0.79$, $P<0.001$). Thus even though the groups varied somewhat in terms of representation of patients, relatives and professionals there was considerable consistency in what members within different groups expected to gain from their membership. There were, however, some significant variations across groups in the extent to which certain specific expectations were held.

Although providing support for cancer patients and their families was among the most salient of expectations within all groups, the mean rating for this expectation varied significantly ($P<0.01$) across groups from 1.0 (groups 1, 5, 7, 8) to 1.6 (group 2). Similarly, being free to express their feelings openly was considered more important ($P<0.001$) in group 7 (mean rating = 1.5) than in group 3 (mean rating = 2.5) while fund raising for Tak Tent was considered more relevant ($P<0.001$) by the members of group 1 (mean

**Table V** Mean ratings of expectations (Exp.), satisfactions (Sat.) and means of differences (Diff.) between them

| Expectation | | Mean ratings Pats. | Rels. | Profs. | Others | P |
|---|---|---|---|---|---|---|
| To meet other people going through or have gone through similar experiences to myself because of cancer | Exp. | 1.24 | 1.56 | 2.44 | 2.70 | 0.001 |
| | Sat. | 1.83 | 1.60 | 3.23 | 3.40 | 0.001 |
| | Diff. | 0.65 | 0.42 | 0.75 | 0.78 | NS |
| To give support to cancer patients and their families | Exp. | 1.48 | 1.27 | 1.10 | 1.00 | 0.008 |
| | Sat. | 2.83 | 2.39 | 1.96 | 2.45 | NS |
| | Diff. | 1.34 | 1.00 | 0.86 | 1.45 | 0.003 |
| To be free to express my own feelings openly | Exp. | 1.65 | 1.68 | 1.97 | 1.92 | NS |
| | Sat. | 1.88 | 1.86 | 1.93 | 1.40 | NS |
| | Diff. | 0.23 | 0.15 | 0.00 | −0.50 | NS |
| To make new friends | Exp. | 1.67 | 1.46 | 2.07 | 1.33 | 0.001 |
| | Sat. | 1.81 | 1.35 | 1.48 | 1.45 | 0.004 |
| | Diff. | 0.19 | −0.09 | −0.59 | 0.09 | NS |
| To provide practical help for cancer patients and their families | Exp. | 1.72 | 1.33 | 1.47 | 1.25 | NS |
| | Sat. | 3.33 | 2.77 | 2.54 | 3.27 | 0.004 |
| | Diff. | 1.51 | 1.44 | 1.07 | 2.09 | NS |
| To find out more about cancer and its treatments | Exp. | 1.73 | 1.43 | 2.20 | 1.67 | 0.003 |
| | Sat. | 2.12 | 1.57 | 2.47 | 1.80 | 0.004 |
| | Diff. | 0.40 | 0.16 | 0.27 | 0.00 | NS |
| To learn from others about how to cope better with cancer | Exp. | 1.82 | 1.32 | 1.53 | 1.33 | 0.009 |
| | Sat. | 2.46 | 2.00 | 2.10 | 2.18 | NS |
| | Diff. | 0.64 | 0.70 | 0.57 | 0.82 | NS |
| To help raise money for Tak Tent | Exp. | 1.87 | 1.65 | 2.28 | 1.42 | 0.003 |
| | Sat. | 2.44 | 2.02 | 2.53 | 2.36 | NS |
| | Diff. | 0.58 | 0.35 | 0.28 | 0.91 | NS |
| To be able to talk about my problems and be listened to by the members | Exp. | 1.90 | 1.61 | 2.77 | 2.58 | 0.001 |
| | Sat. | 2.78 | 2.38 | 3.43 | 3.82 | 0.007 |
| | Diff. | 0.79 | 0.81 | 0.67 | 1.09 | NS |
| To get support from other members of the group to help me to cope better with my own/my relative's cancer | Exp. | 1.96 | 1.55 | 2.93 | 3.00 | 0.001 |
| | Sat. | 2.30 | 2.02 | 3.61 | 3.70 | 0.001 |
| | Diff. | 0.30 | 0.48 | 0.67 | 0.70 | NS |
| To give other members advice on how they can cope better with cancer | Exp. | 2.17 | 2.52 | 1.79 | 3.33 | 0.02 |
| | Sat. | 3.02 | 3.17 | 2.62 | 3.73 | 0.001 |
| | Diff. | 0.85 | 0.63 | 0.83 | 0.27 | NS |
| To be able to tell other group members things in confidence that I would not tell people outside the group | Exp. | 2.21 | 1.66 | 2.71 | 2.92 | 0.001 |
| | Sat. | 3.13 | 2.50 | 3.68 | 3.80 | 0.004 |
| | Diff. | 0.86 | 0.78 | 0.92 | 0.90 | NS |

rating = 1.29) than by those in group 4 (mean rating = 2.8). Thus while there was general agreement across groups on what a cancer support group might be expected to provide for its members there were nevertheless differences in the emphasis accorded to particular activities.

Analysis of the relative satisfaction of expectations across individual groups also showed a significant level of consistency ($W = 0.46$, $P < 0.001$). That is, the relative extent to which the different expectations were being fulfilled was found to be similar across individual groups. In addition, analysis of variance confirmed that there was no significant variation among the groups in terms of the overall degree to which they were meeting their members' expectations ($P > 0.2$). Taken together with the results discussed in the previous paragraph, therefore, these findings indicate that there was very little difference among Tak Tent's individual groups both in the expectations held by members and in the relative degree to which these were being met.

### Discussion

The design of the present investigation clearly does not permit any definitive conclusions to be drawn about the potential benefits of cancer support organisations such as Tak Tent. Indeed since the Tak Tent membership is entirely self-selected and each group functions somewhat differently it would be extremely difficult to conduct any sort of randomised, controlled evaluation of the groups' efficacy. It is not possible to say, therefore, whether membership of such an organisation actually does enable patients or relatives (or staff) to cope better with cancer, or whether there may even be adverse consequences for some people who join. Rather the nature of the current study has been largely descriptive and limited to evaluating the views of those who voluntarily chose to belong to the groups. Notwithstanding the limitations of this approach, however, the results obtained do provide an insight into the characteristics of those who decided to attend Tak Tent and into their reasons for joining.

In so doing, they point to two main conclusions. First, although Tak Tent was open to all cancer patients and their families, its membership seemed limited in particular respects. Second, while the Tak Tent groups appeared to be largely meeting the principal expectations of their members some aspects of the group experience seemed to be less satisfying than others. The implications of these observations merit further consideration.

### (a) *Limitations on membership*

A striking feature of Tak Tent's membership was the preponderance of middle-aged women from the higher social classes and the comparative exiguity of the young, men and lower social classes. Since this has also been found to hold for similar organisations studied in the US (Johnston & Stark, 1980; Maisaik *et al.*, 1981; Falke & Taylor, 1983), it appears that cancer support groups generally attract a relatively small proportion of the population which they seek to aid. Why is this?

While the apparent age bias may simply reflect the greater incidence of cancer among older people, the dominance of women and the higher social classes are not so readily explained. However some possibilities can be postulated.

Differences in the socialization of the sexes may be important. In Western cultures males are expected to contain their emotions more than women and may therefore be less willing to participate in groups where feelings are shared openly. Moreover, the traditional role of females as carers in society may make women more likely to join support groups purely to offer, as opposed to seeking, help. A further factor may also operate in the case of patients. The cancer which most commonly afflicts women (i.e. breast cancer) has a longer natural history than that which most frequently affects men (i.e. lung cancer). Thus more female patients than male patients may find themselves having to cope with the problems associated with cancer for an extended period and, in this circumstance, may perceive greater benefit in joining a support group.

That people from social classes IV and V tend not to participate in cancer support groups now seems established but the reasons for this remain a matter of conjecture. It is possible that difficulty in articulating their own needs and feelings inhibits some from joining groups where others are more able to express themselves. Alternatively there may be variations in the way in which different social classes respond to cancer. There is some evidence to suggest that people of lower social class hold a more fatalistic view of illness than do those from higher classes (Pill & Stott, 1985). The lack of involvement of the former in support organisations may therefore reflect a tendency to react more passively to cancer.

Whatever the explanation for the evident biases in Tak Tent membership, however, it seems certain that the problems precipitated by cancer are no less common among those sections of society which currently do not use support groups than among those which do. If such groups can indeed assist some people to adjust better to cancer, therefore, future research must elucidate why their appeal is limited and suggest how it might usefully be extended.

### (b) *Fulfilment of members' expectations*

The results suggest that Tak Tent members both expected and found their groups to be 'safe' environments within which they could express their own feelings openly. Similarly, the development of friendships was a widely anticipated outcome of group participation which seemed generally to have been realised. That the professionals exceeded their expectations of making friends perhaps suggests that they initially expected their professional status somehow to set them apart but found that conventional distancing between 'professionals' and 'clients' did not obtain within the groups.

The patients and relatives expected groups to enable them to meet others enduring similar experiences to themselves and seemed fairly content that this view had been confirmed. There was also an expectation that Tak Tent would have an educational role which appeared to have been largely fulfilled, presumably because the organisation produces information booklets for members, and groups frequently invite guests to talk on various cancer related topics.

What members appeared rather less satisfied with, however, was the extent to which group participation had enabled them to learn how to cope better with cancer, to share their problems and to provide help for others. This is disappointing since these are held to be among the most potentially beneficial aspects of mutual support groups. It has been argued that groups afford a good opportunity for people to learn new coping strategies by either modelling the behaviour of, or gaining ideas from, other members (Gussow & Tracy, 1976). Similarly, for those whose existing social networks are limited or ineffective such groups have been seen by many as an important source of social support, offering alternative social contacts with whom problems may be shared (Bloom, 1982). In addition, it has been suggested that a signal feature of support groups is that they enable individuals to help others. This not only benefits those who are aided, but, according to the helper-therapy principle espoused by Reissman (1976), may be more therapeutic for those who do the helping.

It is not clear why Tak Tent was apparently fulfilling these functions less effectively than others. Perhaps it was because many groups were in the early stages of development and had not progressed to a point where members felt able to share problems with each other. Alternatively, members may have been unsure of how to go about supporting each other. It is perhaps relevant that the professionals, presumably with training in helping clients were significantly more satisfied with the support they had given within the groups than were the lay members. It is conceivable, therefore, that some basic training in skills like listening and counselling might serve to enable group participants to feel more confident in giving support. Such training is now offered to Tak Tent members and its impact on the effectiveness of the groups remains to be seen.

Thanks are due to Prof. Kenneth C. Calman for help and advice in setting up the study and for useful comments on this paper. The contribution made by Mrs Jean Morrison and Mrs Sylvia Cochrane to the research is also gratefully acknowledged as is the financial support provided by the Cancer Research Campaign.

Finally we would especially thank the members of Tak Tent for their co-operation and assistance in conducting the survey.

### References

BLOOM, J.R. (1982). Social support systems and cancer: A conceptual view. In *Psychological aspects of cancer*, Cohen, J. *et al.* (eds). Raven Press: New York.

BROWN, T. & GRIFFITHS, P. (1986). Cancer self help groups: An inside view. *Br. Med. J.*, **292**, 1503.

FALKE, R.L. & TAYLOR, S.E. (1983). Support groups for cancer patients. *UCLA Cancer Centre Bull.*, Fall: 13.

FRIEDENBERGS, I., GORDON, W., HIBBARD, M., LEVINE, L., WOLF, C. & DILLER, L. (1981). Psychosocial aspects of living with cancer: A review of the literature. *Int. J. Psych. Med.*, **2**, 303.

GUSSOW, Z. & TRACY, G.S. (1976). The role of self-help clubs in adaptation to chronic illness and disability. *Soc. Sci. Med.*, **10**, 407.

JOHNSTON, E.M. & STARK, D.E. (1980). Cancer patients and their family members in an acute care teaching hospital. *Social Work Hlth Care*, **5**, 335.

KILLILEA, M. (1976). Mutual help organisations: Interpretations in the literature. In *Support systems and mutual help: Multidisciplinary explorations*, Caplan, G. & Killilea, M. (eds). Grune and Stratton: New York.

MAISAIK, R., CAIN, M., YARBRO, C.H. & JOSOF, L. (1981). Evaluation of TOUCH: An oncology self-help group. *Oncol. Nurs. Forum*, **8**, 20.

NIE, N.H., HULL, C.H., JENKINS, J.G., STEINBRENNER, K. & BENT, D.H. (1975). Statistical package for social sciences. McGraw-Hill: New York.

PILL, R. & STOTT, N.C.H. (1985). Choice or chance: Further evidence on ideas of illness and responsibility for health. *Soc. Sci. Med.*, **20**, 981.

REISSMAN, R. (1976). How does self-help work? *Social Policy*, 41.

TELCH, C.F. & TELCH, M.J. (1985). Psychological approaches for enhancing coping among cancer patients: A review. *Clin. Psychol. Rev.*, **5**, 325.

WEBB, P.A. (1983). Ready, willing but able? – The self help group. *J. Royal Soc. Hlth*, 35.

WEIR, J., DEANS, G. & CALMAN, K.C. (1985). Tak Tent – a practical approach to patient, relative and staff support. In *Psychological aspects of cancer*, Watson, M. & Morris, T. (eds). Pergammon Press: Oxford.